# National survey focusing on the crucial information needs of intensive care charge nurses and intensivists: same goal, different demands

**DOI:** 10.1186/1472-6947-13-15

**Published:** 2013-01-29

**Authors:** Heljä Lundgrén-Laine, Elina Kontio, Tommi Kauko, Heikki Korvenranta, Jari Forsström, Sanna Salanterä

**Affiliations:** 1 Department of Nursing Science, University of Turku, Turku, Finland; 2 Bureau of Administration, Turku University Hospital, Turku, Finland; 3 Faculty of Telecommunication and e-Business, Turku University of Applied Sciences, Turku, Finland; 4 Department of Biostatistics, University of Turku, Turku, Finland; 5 The Finnish Medical Association, Helsinki, Finland

## Abstract

**Background:**

Although information technology adequately supports clinical care in many intensive care units (ICUs), it provides much poorer support for the managerial information needed to coordinate multi-professional care. To gain a general view of the most crucial multi-professional information needs of ICU shift leaders a national survey was conducted, focusing on the information needs of charge nurses and intensivists.

**Methods:**

Based on our previous observation study an online survey was developed, containing 122 information need statements related to the decision-making of ICU shift leaders. Information need statements were divided into six dimensions: patient admission, organisation and management of work, allocation of staff and material resources, special treatments, and patient discharge. This survey involved all ICU shift leaders (n = 738) who worked in any of the 17 highest level ICUs for adults in university hospitals in Finland during the autumn of 2009. Both charge nurses’ and intensivists’ crucial information needs for care coordination were evaluated.

**Results:**

Two hundred and fifty-seven (50%) charge nurses and 96 (43%) intensivists responded to the survey. The consistency of the survey was found to be good (Cronbach’s α scores between .87–.97, with a total explanatory power of 64.53%). Altogether, 57 crucial information needs for care coordination were found; 22 of which were shared between shift leaders. The most crucial of these information needs were related to organisation and management, patient admission, and allocation of staff resources. The associations between working experience, or shift leader acting frequencies, and crucial information needs were not statistically significant. However, a statistically significant difference was found between the number of ICU beds and the ICU experience of charge nurses with information needs, under the dimension of organisation and management of work. The information needs of charge nurses and intensivists differed. Charge nurses’ information needs related to care coordination, were more varied, and concerned issues at a unit level, whereas intensivists focused on direct patient care.

**Conclusions:**

The reliability and validity of our survey was found to be good. Our study findings show that care coordination at an ICU is a collaborative process among ICU shift leaders with multiprofessional information needs related to organisation and management, patient admission, and allocation of staff resources. Study findings can be used to identify the most crucial information needs of ICU shift leaders when new information technology is developed to support managerial decision-making during care coordination.

## Background

Within intensive care units (ICUs) the coordination of daily care is usually managed by charge nurses and intensivists. The main tasks of the shift leaders are related to ensuring the smooth flow of activities, and a consistent quality of care. Care coordination in ICUs is a complex process that involves coordination of both resources and of patient care [1]. Previous studies have shown that multidisciplinary teamwork in shift leading has an impact on patient safety and work efficiency [2,3]. From an international perspective there has been great interest in the information needs related to clinical work [4-6] as well as team work [7,8]. However, most of these studies have focused on direct care and patient-related tasks [9-11], and have not explored the information needs on the level of the shift leaders. Compared to bedside workers, shift leaders in ICUs need different kinds of information for their decision-making, including indicators that describe and integrate real-time performances and overall situations within the unit. In addition, shift leaders’ roles in care coordination vary; consequently, they have varying multiprofessional information needs [1,12,13]. This should be taken into account when information integration systems are developed to support ICU shift leaders’ daily care coordination. At the moment commercial information systems used in ICUs are still too inflexible regarding the data extraction needed for daily care coordination [14].

The first clinical information systems for ICU patient care were introduced in the early 1970s [15]. In Finland today all hospitals in both the public and private sector use electronic patient record systems [16], but information management and enterprise management systems for shift leaders are still at an embryonic stage. Currently, new technologies have enabled information integration and formation within units. However, most of these stand-alone patient care systems in ICUs are developed to support only direct patient care, and are not integrated with other systems used in the unit or at a hospital level.

In many units the limitations of these information systems have forced shift leaders to create their own information tools to support their information integration. Typically these clinician-designed tools - such as to do lists, single-use worksheets, or different kinds of white boards - are developed only for temporal use, and require pieces of information to be manually gathered from multiple sources [12,17-19]. Previous studies have shown that patient mortality outcomes are associated with delays in ICU admissions, readmissions, unplanned night discharges, and poorly organised transfers between hospital units [20-23]. Typically, many of these decisions are made by the shift leaders in ICUs with the information available at the present moment, and are often primarily reliant on memory. However, we currently do not know how information integration, and support for real-time information access, could impact on ICU patient outcomes.

Presumably, fast and easy access to clinical and managerial information plays a vital role in ICU shift leaders’ decision-making. In this paper we are specifically concentrating on the most crucial information needs of ICU shift leaders during daily-care coordination, as our ultimate goal is to develop information management tools to support ICU shift leaders’ decision-making. For this reason we here are aiming to gain a better understanding of what information should be immediately accessible, and what crucial information is needed by both the charge nurses and intensivists.

The following research questions are thus central to this study:

1. How well do our survey statements capture the most crucial information needs of ICU shift leaders during daily-care coordination?

2. What are the most crucial information needs of ICU charge nurses and intensivists during the coordination of daily care?

3. Do the most crucial information needs differ between the ICU charge nurses and intensivists?

## Methods

### Research setting

Finland has a population of approximately 5.4 million, and the mainland is divided into 20 hospital districts, which vary in population from 740,000 to nearly two million. Each hospital district has a main central university hospital, resulting in five university hospitals in total, which are responsible for coordinating and providing specialised health care services for the district population, including the highest level of care (LOC III, [24]) for the most challenging intensive care patients.

This nationwide study was conducted between September and November 2009, and covered all ICUs for adults in Finland (n = 17) that deliver the highest level of care. Each hospital district authority approved the study protocol, and the Ethical Committee of the Hospital District of Southwest Finland also conducted an ethical evaluation (Satakunta Hospital 9/2009). All of the study units have an academic teaching mission, and the required organisational characteristics: specifically that the ICU is able to deliver the highest level of care, that they are close model units [25], and that they have an appointed charge nurse and intensivist in every shift.

Before the survey, the unit consultants and head nurses were sent a short study outline explaining our inclusion criteria and our definitions of charge nurses’ and intensivists’ job descriptions. These outlined that intensivists in charge should have the overall responsibility of the units and patient care, and that charge nurses should be assigned as shift leaders on a particular shift, and be responsible for coordinating nursing activities (for example, ensuring staff allocation and staff skill mix, patient admission and discharge facilitation, and material and equipment availability).

All the participating units used stand-alone information systems. None of the units had a specific computer programme for the shift leaders. The number of ICU beds varied from five to 24, and the largest units had approximately two thousand admissions per year. The proportion of charge nurses and intensivists varied between the participating ICUs; however, in two-thirds of the units there was an equal number of charge nurses and intensivists who worked as shift leaders, whereas in one-third of the units there were almost three times the number of charge nurses compared with intensivists.

### Online survey development and implementation

The online survey (Webropol®) conducted in this study is based on our previous observational study where we used a think-aloud technique and protocol analysis to identify the ad hoc decision-making of ICU shift leaders. Ad hoc decisions were defined as critical judgements that are needed for a specific purpose at a precise moment, with the goal of ensuring instant and adequate patient care and a fluent flow of ICU activities [26]. Each ad hoc decision found in the observation study was combined with an information need, which altogether constituted 122 statements related to the ICU shift leaders’ immediate information needs.

Before the final survey, 13 people with experience in nursing, medicine, or computer science evaluated our questionnaire for the clarity of the questions, the intelligibility of the concepts and the scale used, as well as the technology of the online program. We also measured the time it took them to respond, and the average response time was under 20 min. The survey was also piloted in a mixed medical 12-bed ICU that fulfilled the study inclusion criteria.

Prior to beginning the survey, the local ICU management was informed. The managers of the units, department head nurses, and medical officers acted as study coordinators and provided the e-mail addresses of the shift leaders. All of the charge nurses (n = 515) and intensivists (n = 223) working in the 17 study units during the data collection were recruited for the study. Finnish ICU nurses do not have any specific education for the position of charge nurse, which means that (depending on the opinion of the unit head nurse) all ICU nurses who have at least one year of ICU experience will usually work as a charge nurse at some time. The number of ICU charge nurses varied between the units, from 15 to 73. A request to participate in the survey and an information letter was sent to each participant, which included a personal online survey link. The respondents were able to respond to the survey anonymously, online, whenever and wherever they wanted; it was not dependent on a certain web browser, and they could also suspend the survey to continue it at a later time. Answering the online survey was considered as informed consent for participation.

Our final survey was composed of two parts: (i) the demographics of the respondents, and two background questions concerning the characteristics of the unit; and (ii) 122 statements divided into the following six dimensions regarding information needs related to the daily activities of the ICU:

• Patient admission (21 statements)

• Organisation and management of work (60 statements)

• Allocation of staff (18 statements)

• Material resources (7 statements)

• Special treatments (5 statements)

• Patient discharge (11 statements).

With each statement, the necessity of the information needed was measured on a rating scale from 0 (completely unnecessary) to 10 (absolutely necessary). All statements were in the same format, such as ‘immediate information about patient’s name is…’ or ‘immediate information about real-time workload at the unit is…’.

### Statistical analysis

Descriptive statistics such as frequencies, percentages, means, medians, and standard deviations (SD) were used to summarise the data. The statistical analysis of the data was performed using SAS 9.2 [27].

To answer the first research question, a confirmatory factor analysis was conducted. This confirmatory factor analysis was used to verify the construct of the survey, which was based on our previous observation study about the ad hoc decision-making of ICU shift leaders. Sum-variables were constructed from the above-mentioned six dimensions of daily ICU activity (patient admission, organisation and management of work, allocation of staff, allocation of material resources, special treatments and patient discharge). Cronbach’s α scores were calculated for each of these six dimensions to verify the internal consistency of the scales. Confirmatory factor analysis was performed with iterated principal factor analysis to extract the factors, using the ASMC prior communality estimation method with orthogonal Varimax rotation.

To answer the second research question we used median values. Median values were chosen because the distribution of the data was distorted, due to the nature of the ordinal scale. To identify the absolutely necessary information needs of ICU shift leaders, the 10-point scale was divided into three sections; responses with a median lower than 5 were considered ‘not important’, responses with a median 5–8 were regarded ‘important’, and the responses with a median of 9–10 were considered as ‘absolutely necessary’ information. Frequencies and percentage values were then calculated from those responses with a median of 9–10. At this point, a cut-point was set at 70%, meaning that if 70% of the respondents rated the statement as absolutely necessary (median 9–10) then it was considered as the most crucial information needed. The level was determined by frequency plots, and by finding the most suitable change-point, where only a quarter of respondents did not consider the statement absolutely necessary. By using a restrictive 70% cut-point, this study attempted to limit the volume of the results to enable the actual discovery of the most crucial information needs of ICU shift leaders, and thus be able to more easily generalise the findings.

To answer the third research question the respondents were divided into four groups depending on age (25–34 yrs, 35–44 yrs, 45–54 yrs and 55–63 yrs), and into four groups depending on the length of work experience in the ICU (0–5 yrs, 6–10 yrs, 11–15 yrs and over 16 yrs). This classification then allowed us to investigate the relationships between age, work experience, and crucial information needs. The Wilcoxon rank-sum test (Mann–Whitney U) was used to find out the differences in information needs between charge nurses and intensivists, and reveal the differences between the sum-variables and the classifying variables of education and in-charge-position frequency. Other non-parametric tests, such as the median test and the Kolmogorov-Smirnov test, were used to support the Wilcoxon test. After performing variable transformation and confirming the normality with simulation from a known distribution, the parametric ANOVA models were fitted for each sum-variable. The hospital was defined as a random effect, and all classifying variables with first-, second-, and third-level interactions were defined as fixed effects.

**Figure 1:**
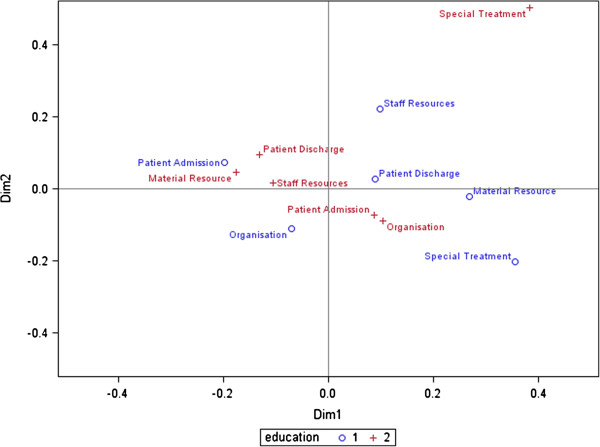
Interactions between six dimensions and differences between charge nurses, o 1 (blue) and intensivists, + 2 (red).

The final model selection was done using stepwise modelling and by comparing Akaike Information Criteria (AIC). To obtain the correct estimates, the degrees of freedom were adjusted with the Satterthwaite method. The least-square means were adjusted with the Tukey method. Pearson’s chi-square test was used to determine the differences between the individual responses of the charge nurses and the intensivists. It showed the equality of frequency distributions if a statement was considered most crucial or not. Finally, correspondence analysis was performed to visualise the interactions between the six dimensions and the differences between the nurses and intensivists in a single scatter plot (Figure 1). A 5% significance level was used throughout the analysis.

## Results

### Respondents

The speciality areas of the study ICUs differed, and included mix medical ICUs, postoperative surgical ICUs and one ICU specialising in burns; however, all of the units were third level ICUs for adults. A total of 515 charge nurses and 223 intensivists were found eligible for the study during the data collection. The overall response rate was 47.8% (n = 353). The response rate was 50% (n = 257) for the charge nurses and 43% (n = 96) for the intensivists, thus the rates of this study were consistent with those seen in recent multi-professional surveys conducted in ICUs e.g. [28]. Table 1 shows the demographic data of the participants.

**Table 1:** Demographic data of the participants

	**Charge nurses**	**Intensivists**
**n**	257	96
**Sex**		
Male	38 (15%)	49 (51%)
Female	219 (85%)	47 (49%)
**Age, years**		
Min, max	25, 61	29, 63
Mean, SD	43, 8.6	43, 7.3
Not reported	1	
**Age groups**		
25–34	20%	10%
35–44	39%	46%
45–54	32%	36%
55–63	9%	8%
**Experience in ICU years**		
Min, max	1, 35	0, 30
Mean, SD	15, 7.9	10, 6.6
Not reported		2
**Shift leader’s duties**		
More than once a week	23.3%	29.2%
On average one a week	26.4%	22.9%
2–3 times a month	28.3%	33.3%
Less often	22.0%	14.6%

*Min* – Minimum; *Max* – Maximum; *SD* - standard deviation.

### Validation and reliability of the survey

To find out how well our survey statements capture the most crucial information needs of ICU shift leaders during the management of daily care, both the face validity and usability of our survey were pre-tested. The Cronbach’s α scores were calculated, and a confirmatory factor analysis was conducted to confirm the consistency of the survey. Cronbach’s α scores for the components of each of the six dimensions were as follows: patient admission (.87), organisation and management of work (.97), allocation of staff resources (.96), allocation of material resources (.91), special treatments (.87), and patient discharge (.90). The confirmatory factor analysis supported these six dimensions, and the total explanatory power was 64.53%. This means that the hypothesised dimension model in our survey matched, and we were thus able to capture the phenomenon fairly well see e.g. [29]. The explanatory power of each factor varied between 2.82% and 21.13% (patient admission, 10.52%; organisation and management of work, 21.12%; allocation of staff resources, 21.13%; allocation of material resources, 2.83%; special treatments, 4.64%; patient discharge, 4.29%). Table 2 presents the factor loadings for each crucial information need. The overall Kaiser's MSA was 0.93, and can thus be considered acceptable.

**Table 2:** Fifty-seven most crucial information needs of ICU shift leaders (n = 353) found under the six dimensions

**Crucial information needs of ICU shift leaders under six dimensions**	**n (%)**	**Dif. %**	***p*-value**	**Factor loadings**
**Admission (n = 10)**				
Need to isolate the patient	**341 (97)**	8.2	<0.001	**0.60**
Patient’s need for mechanical ventilation	**297 (84)**	11.1	NS	0.58
Method of patient isolation	**287 (81)**	10.1	NS	0.48
Number of planned patients	**247 (70)**	11.7	NS	0.32
Urgency of the patient’s condition	245 (69)	4.8	NS	0.36
Patient’s personal identity code	231 (65)	22.6	<0.001	0.44
Emergency operations	222 (63)	12.0	NS	0.45
Patient’s scheduled time of arrival at the ICU	218 (62)	**37.6**	<0.001	0.52
Patient’s diagnosis	213 (60)	11.6	NS	0.45
Criterion/criteria for patient’s admission to the ICU	204 (58)	29.4	<0.001	0.46
**Organisation & management (n = 24)**				
Patient’s death	**309 (88)**	1.5	NS	0.31
Number of patients on the unit	**298 (84)**	23.0	<0.001	0.53
Special treatments given to patients	**293 (83)**	11.0	NS	0.59
Removal of a patient from isolation	**278 (79)**	18.0	<0.001	0.65
Patients admitted to ICU	**273 (77)**	16.1	<0.001	**0.65**
A significant change in the patient’s condition during one’s shift	**270 (76)**	12.1	NS	**0.63**
Staff skills and knowledge	**269 (76)**	**40.3**	<0.001	**0.61**
Scheduled examinations that will require patient transfer	**259 (73)**	**36.3**	<0.001	0.58
Normal staffing levels for each shift	**257 (73)**	**34.2**	<0.001	0.53
Staff sick leave	**248 (71)**	**54.0**	<0.001	0.52
Number of patients per room	244 (69)	**44.9**	<0.001	0.42
Staff on duty	239 (68)	**50.1**	<0.001	**0.64**
Patient medication that requires intensive monitoring	238 (67)	1.0	NS	0.54
Compulsory infection samples	236 (67)	23.2	<0.001	0.53
Real-time workloads at the unit	228 (65)	22.9	<0.001	0.43
Special skills of nursing staff	220 (62)	**48.4**	<0.001	0.59
Staff induction needs	216 (61)	**54.0**	<0.001	0.53
Patients presented for admission to ICU	198 (56)	3.1	NS	0.47
Complications arising during intensive care	193 (55)	6.5	NS	0.44
Patient’s will to live	192 (54)	14.0	NS	0.53
Nursing staff skill mixes	191 (54)	**58.6**	<0.001	0.53
Roster plans	185 (52)	**42.0**	<0.001	0.55
Patient’s care intensity	179 (51)	**41.0**	<0.001	0.52
**Allocation of staff resources (n = 9)**				
Nurse in charge/physician in charge of the unit	**270 (76)**	29.2	<0.001	0.58
Staffing level on current shift	**258 (73)**	**50.3**	<0.001	**0.77**
Staff resources that can be released (e.g. for transport)	**253 (72)**	**45.5**	<0.001	**0.77**
Number of nursing staff per patient	**248 (71)**	**39.3**	<0.001	**0.71**
Staffing levels for scheduled rosters	239 (68)	**42.9**	<0.001	**0.76**
Number of nursing staff per patient room	236 (67)	**41.8**	<0.001	**0.71**
Staff working on next shift	231 (65)	**58.4**	<0.001	**0.85**
Skill mix on current shift	230 (65)	**56.6**	<0.001	**0.82**
Patient’s nurse on each shift	212 (60)	**42.4**	<0.001	**0.71**
**Special treatments (n = 5)**				
Planned special treatments	**310 (88)**	11.9	0.002	0.56
Scheduled dates for surgery or procedures	**284 (80)**	11.8	NS	0.58
Start time of special treatments	240 (68)	20.4	<0.001	0.50
End time of special treatments	195 (55)	12.9	NS	0.38
Special treatment consultation responses	186 (53)	9.2	NS	0.49
**Allocation of material resources (n = 3)**				
Vacant beds at the unit	**276 (78)**	15.8	<0.001	0.30
Products needed for special treatments	219 (62)	23.7	<0.001	0.42
Fixed equipment around each bed on the unit	178 (50)	**33.5**	<0.001	0.54
**Discharge (n = 6)**				
Patient being discharged	**277 (78)**	26.2	<0.001	0.59
Transport cancellations	**256 (73)**	26.6	<0.001	0.50
Patient’s time of discharge	240 (68)	**37.6**	<0.001	**0.64**
Planned time of transport	217 (61)	**44.4**	<0.001	**0.68**
Staff resources available for transport	154 (44)	**34.9**	<0.001	**0.62**
Ward where the patient is going to be/has been notified	191 (54)	14.2	NS	0.44

Shared information needs, largest differences, and highest factor loadings are highlighted in bold text.

### The most crucial information needs for ICU shift leaders

To discover the most crucial information needs of ICU shift leaders we first observed the absolutely necessary information (median values of 9–10). Subsequently, the information that was rated as absolutely necessary by 70% of all the respondents was considered the most crucial (cut-point of 70%). The analysis revealed that there were a total of 57 crucial information needs out of a potential 122. These information needs covered all six dimensions: patient admission, organisation and management of work, allocation of staff resources, allocation of material resources, special treatments, and patient discharge (Table 2).

For the second research question the shared multi-professional crucial information needs were identified. In total, 22 shared crucial information needs were found (highlighted in Table 2), most of these were related to patient admission, organisation and management of work, or resource allocation. The most emphasised shared crucial information needs were related to direct patient care, such as the need to isolate the patient (mean 9.7, SD 1.2), the death of a patient (mean 9.4, SD 1.5), planned special treatments (mean 9.4, SD 1.4), a patient’s need for mechanical ventilation (mean 9.3, SD 1.5), special treatments given to patients (mean 9.2, SD 1.7), the number of patients in the unit (mean 9.2, SD 1.8), and the method of patient isolation (mean 9.2, SD 1.8). Nearly 90% of all ICU shift leaders reported that immediate information about the need to isolate the patient, the death of a patient, and planned special treatments were absolutely necessary for their ad hoc decision-making during the management of daily activities. The highest factor loadings were found regarding issues related to allocation of staff resources (see Table 2).

Statistically, these crucial information needs were not significantly associated with age, length of working experience, or the shift leader acting frequencies of charge nurses or intensivists. However, a statistically significant difference was found between both the number of ICU beds and the length of ICU experience of charge nurses, and information needs under the dimension of organisation and management of work (ANOVA). Differences were found among ICUs with bed numbers <10 or 10–15, and ICUs with bed numbers >20 (p = 0.003, p = 0.01). The number of ICU beds also seems to impact upon information needs related to special treatments and patient discharge. In addition, the responses of charge nurses with 5–10 years of work experience differ from those with other levels of work experience in questions relating to the dimension of organisation and management of work, as a statistically significant difference (p = 0.02) was found for responses related to the dimension of organisation and management of work between charge nurses with less than 5 years work experience and those with 5–10 years.

**Figure 2:**
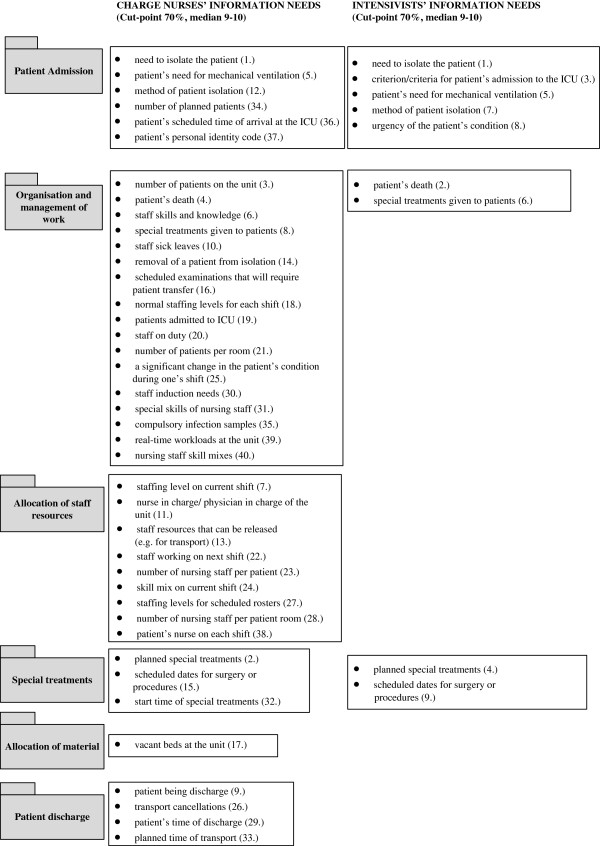
**The most crucial information needs of charge nurses and intensivists.** (Order of importance marked inside the brackets).

For the third research question the most crucial information needs were considered separately for charge nurses and intensivists, although the cut-point was maintained at 70%. It was foreseeable that information needs between nurses and physicians differ. However, previous research has not shown in which areas these differences occur. Interactions between six dimensions and differences between charge nurses are shown in Figure 1. On the whole, ICU charge nurses rated a higher number of information needs as crucial than intensivists. Altogether, charge nurses evaluated 40 out of 122 information needs to be the most crucial (which are presented in order of importance under each dimension in Figure 2: the numbers inside the brackets represent the order of importance of these 40 most crucial information needs).

Our analysis revealed that the most crucial information needs of charge nurses were related to the organisation and management of work, and allocation of staff resources, whereas the most crucial information for intensivists concerned admission, and special treatments. The biggest differences between nurses and intensivists concerned staff related issues and timings of activities, which were emphasised more frequently by the charge nurses (Table 2).

## Discussion

The main goal of our study was to discover the most crucial information needs of ICU shift leaders that support the coordination of daily care in their fast based units. Our study showed that ICU shift leaders need a wide variety of crucial information for their managerial decisions. Furthermore, the information needs of ICU charge nurses seem to be focused more on the unit level, whereas intensivists concentrate on direct patient care. Most of the crucial information needs of intensivists were related to direct patient care, whereas charge nurses needed more information about resource allocation, and issues concerning organisation and management. These findings are important when information management tools are developed for ICU shift leaders, as different crucial needs would inevitably require different tools to support them. Significantly, to date, only a few studies have focused on multi-professional information needs in ICUs e.g., 1, [30], and thus these differences are currently underexplored. Furthermore, the focus in previous studies has been on direct patient care, rather than on the entire process of the management of multi-professional activities within ICUs.

We found that the most crucial information needs of ICU shift leaders focused on two areas: one is the state of a patient, especially during the admission phase, including the need to isolate, the need for mechanical ventilation, the urgency level, and planned special treatments; and two, the constantly changing requirements for adequate resource allocation, such as the number of patients, vacant beds, and staffing levels. The most crucial information needs of charge nurses and intensivists differed, even if their goals – a smooth flow of activities and good quality patient care – were the same. The shift leaders also stressed that both the critical state of patients and inadequate resource allocation greatly impact the workflow management of an ICU. However, clearly, both charge nurses and intensivists prioritised the information needs that were essential for ensuring the best possible patient care; only after this was achieved did they address issues related to ensuring sufficient resources in every situation.

Unlike a previous study by Gurses et al. [12], which did examine the transfer of information and care coordination, but only from a nurse’s perspective, this study considered both nurses and physicians. However, our results are still supported by previous results [1,12,19]. Gurses et al. [12] found that the information needs of trauma hospital nurse coordinators were related to the patients’ state, and resource allocation issues. For example, nurse coordinators needed information about infection results, isolation needs, special equipment, vacant beds, staffing levels, and terms or plans of patient discharge. In the review by Gurses et al. [19] the information needs of multi-disciplinary rounds were divided into three categories: clinical information, reference information in regards to diagnosis and treatments, and information related to organisational and social issues. The results of this study suggest that the most crucial information needs of intensivists are more closely connected to clinical and reference information, while the most crucial information needs of charge nurses are mainly related to reference and organisational information. The study by Miller et al. [1] presented similar conclusions, dividing care coordination and management in ICUs into two different information spaces: coordination of resources, and coordination of patient care.

Statistical analysis revealed that charge nurses with 5–10 years of work experience differed from the other groups when regarding information needs under the dimension of ‘organisation and management of work’. A statistically significant difference was found for charge nurses with fewer than 5 years of work experience (p = 0.02). Our results can be explained by a previous study by Benner [31] as, according to Benner, nurses with 5–10 years of work experience are able to reflect and modify their decision-making in response to rapidly changing situations. Less experienced workers are more rule-based and inflexible, whereas more experienced workers have an intuitive way of working. Charge nurses with 5–10 years of work experience seem to be more confident in defining their immediate and necessary information needs. In addition, the number of beds had an influence on charge nurse’s information needs. Although it is understandable that the coordination of smaller units is easier than those units with beds numbering over 20, and also that the speciality area of the unit might have an influence on the information needs, similar differences were not found between the intensivists. This therefore may reflect that their information needs, related to direct patient care, are better supported.

For ICU shift leaders, handling all of the information needed by memory is impossible. For decisions related to the care coordination, ICU shift leaders must typically gather all of the information required from disparate sources several times during their shift. Gurses et al. [12] found that nurse coordinators who led patient flow management spent 75% of their round-time exchanging information. Communication with other professionals was the most often-used method of doing this. To be able to control and manage the information flow, nurse coordinators developed a paper-based, non-official summary that they used as an ad hoc information tool. Out of curiosity, our study provided one of the charge nurses with a pedometer, to measure the distance walked when seeking this information; during one morning shift, she walked approximately three-and-a-half kilometres for this purpose, in a unit with an area of 1,100 m^2^.

Evidently, some of the most crucial information found will endure for longer in the future of ICU workflow management and information exchange. Such crucial information needs might include the information needed to isolate the patient, the patient’s need for mechanical ventilation, the urgency of the patient’s condition, planned special treatments, the number of nursing staff per patient, and staff skills and knowledge. However, this study clearly shows that the crucial information needs of ICU professionals differ, and are based on their roles and tasks within the unit. This study, therefore, could also help software companies improve the usability of their software, as different health professionals may need differently organised screens for the same data. Furthermore, the methodology used in this study can be applied to determine the most important aspects of the information, which could then be shown immediately to the user on-screen.

### Limitations and strengths

Our survey is limited to shift leaders in the highest level ICUs, so no generalisations can be made for lower-level units or other professionals working in ICUs. However, the information need statements and study results can be applied to other settings. The survey was conducted in one country and many factors, such as geographical location, cultural elements, specialisation of units, and staff structure or responsibilities may affect what is categorised as the most crucial information for each unit. On the other hand, the study sample was large and the study units successfully represented both charge nurses and intensivists, as well as highest level ICUs with multiple specialities in different parts of the country.

Clearly, the crucial information needs of ICU shift leaders will change over time, and creating a definitive, all-inclusive list of the crucial information needs for ICU shift leaders is impossible. However, the survey used in this study was developed from the findings of our previous observation study [26], in which a list of information needs from the most important ad hoc decision-making areas of ICU shift leaders was constructed after in-depth analysis. The final survey and concepts used were also evaluated and piloted.

The survey was fairly long, as it included 122 statements, yet the nature of the online format made it easy and quick to complete. In addition, using an online survey was a straightforward and economical method for gathering information from a large group of participants, and no technical problems were encountered. In the future, expanding this survey to include international participants, and thus comparing the common information needs of ICU shift leaders globally, would be of interest.

## Conclusions

Managing the daily activities at an ICU is a collaborative process among ICU shift leaders. Our study showed that the most crucial information needs for these shift leaders relate to the organisation and management of work, patient admission, and the allocation of staff resources. Shift leaders greatly emphasised two distinct areas: direct patient care, and resource allocation throughout the ICU stay. Intensivists mainly emphasised information needs related to direct patient care, and charge nurses required information on resource allocation.

The information needs of ICU shift leaders concerning direct patient care are well supported with current information management tools, however a significant gap can be found in the information related to resource allocation. Usually, this information is scattered and must be gathered from different sources, resulting in a time delay even if the information is essential to the outcome of ICU care.

Further research is needed to investigate whether gathering, integrating, and visualising the most crucial multi-professional information needs of ICU shift leaders is possible for the purposes of better supporting and improving the uniformity of the care coordination. This will be even more important if the ever-increasing volume of information, concerning both patient flow and workflow issues, is to be managed effectively when larger multi-specialty units are developed. Traditional, disposable, paper-based information sheets, notes, or tools developed by users cannot ensure good-quality intensive care. Thus, better-developed and flexible real-time information management tools are needed in ICUs to meet the information needs of shift leaders during the coordination of daily activities.

### Availability of survey

A copy of the survey used in this study can be requested from the first author, Heljä Lundgrén-Laine, hklula@utu.fi, at no charge. In published work, citation to this original work is required.

## Competing interests

The authors declare that they have no competing interests.

## Authors' contributions

HLL designed the study, coordinated the online survey and wrote the draft for the manuscript. EK, HK and JF were involved in drafting the manuscript. TK performed the statistical analysis and provided editorial advice. SS participated in the study design as a supervisor and helped to draft and finalise the manuscript. All authors read and approved the final manuscript.

## Pre-publication history

The pre-publication history for this paper can be accessed here:

http://www.biomedcentral.com/1472-6947/13/15/prepub
